# Chop and Change: A Commentary and Demonstration of Classical vs. Modern Measurement Models for Interpreting Latent-Stability of Occupational-Future Time Perspective

**DOI:** 10.3389/fpsyg.2018.01029

**Published:** 2018-06-19

**Authors:** Matthew J. Kerry

**Affiliations:** Department of Management, Technology, and Economics, The Swiss Federal Institute of Technology, Zürich, Switzerland

**Keywords:** measurement, item response theory (IRT), dimensionality, future time perspective (FTP), classical test theory (CTT)

## Abstract

This commentary article was initially motivated by an empirical paper published in the journal of *Work, Aging, and Retirement* that reported support for stability (non-decreasing) future time perspectives (FTP) over two repeated-measurements. That is, empirical evidence supporting the temporal stability of an adapted measure (occupational-FTP [O-FTP]) serves as guiding framework for demonstrating limitations of classical test theory (CTT) and modern psychometrics’ (IRT) enabling extension for stronger substantive inferences from response data. The focal authors’ quantitative attention to study design and statistical analysis is commendable. In this commentary, I aim to complement their efforts from a measurement perspective. This is accomplished through four sections. In the first section, I summarize some well-known limitations to CTT measurement models for assessing change. Then, I briefly introduce item response theory (IRT) as an alternative test theory. In the second section, *Chop*, I review the empirical evidence for FTP and O-FTP’s latent-factor structure. Then, I bring evidence from modern psychometric methods to bear on O-FTP, specifically, a model-comparisons approach was adopted for comparing relative fit of 1-factor, 2-factor, and bifactor solutions in cross-sectional data (*N* = 511). Findings supported retention of the bifactor solution. In the third section, *Change*, I extend the bifactor model to two-wave FTP data over approximately 2 years (*N* = 620) as an instructive application for assessing temporal stability. The fourth section concludes with a brief discussion of substantive implications and meaningful interpretation of (O)-FTP scores over time.

## Introduction

Cronbach and Meehl (1955, p. 288) commented long ago, “Whether a high degree of stability is encouraging or discouraging for the proposed interpretation depends upon the theory defining the construct." Abiding theory, the current commentary is motivated by the necessary integration of three quantitative methodologies, (1) research design, (2) measurement, and (3) data analysis^1^ as informants to research topics (Pedhazur and Schmelkin, 1991). Recently, Weikamp and Göritz (2015) used a longitudinal design and powerful multilevel analysis in their publication on the temporal stability of occupational future time perspective (O-FTP). I aim to complement their publication by emphasizing measurement and its importance when considering phenomenological specificities between work and retirement. The goal is to raise substantive awareness for meaningful interpretation of statistical significance in the research context of aging populations (Kerlinger, 1979). The interchangeability of *statistical* approaches for assessing measurement invariance across groups and over time is a convenient framework toward this goal (Horn and McArdle, 1992; Meredith, 1993).

The remainder of the commentary comprises four sections. In the first section, I summarize notable limitations to assessing change from statistical models applied to measurements based on Classical Test Theory (CTT). In the second section, *Chop*, I review the empirical evidence for factorizing the original FTP instrument and provide new evidence from item response theory (IRT) challenging its justification, including an extension to occupational-FTP. In the third section, *Change*, I extend the same IRT-based model to two- wave FTP data as an instructive application for assessing temporal stability. The fourth section concludes with a brief discussion of substantive implications and meaningful interpretation of FTP stability over time.

## Classical (Ctt) Limitations

Limitations to CTT-based scores for measuring change have been known for some time (Cronbach and Furby, 1970). Less known is that, even latent-variable modeling of change is vulnerable to some statistical artifacts because effect estimation (change) relies on the metric of the observed scores. That is, scores obtain meaning by comparing their position in a norm group which, in turn, makes change scores incomparable when baseline standings differ. This also has implications when, for example, the outcome itself is time-scale dependent (e.g., time or age effects on FTP). In this section, I briefly summarize sources of measurement scale-artifacts that can obscure or mislead researchers when making inferences from longitudinal designs. When warranted, I call attention to specific instantiations of these artifacts in Weikamp and Göritz, as well as analogs between the analytic and measurement perspectives.

### Test Theory Model

If the goal of lifespan research is to study individual differences in change, then relative differences become critical. Pertinent to this understanding is Kerlinger’s (1979) observation, “statistical significance says little or nothing about the magnitude of a difference or of a relation…one must understand the principles involved and be able to judge whether obtained results are statistically significant and whether they are meaningful…” (pp. 318–319). For example, change scores could not be meaningfully compared when baseline levels differ based on ordinal-level scales of measurement, e.g., CTT (Stevens, 1946). Assuming baseline equivalence, differential change (rate-of-change) would be difficult to compare across individuals, as well as non-linear change. In fact, CTT achieves interval-scale properties only by obtaining a normal-score distribution. One could argue, in principle, that the prediction of negative age-related changes in FTP contradicts the distributional assumption required for change-score comparisons.^2^ These arbitrary-metric issues is soluble by IRT’s achievement of interval-level measurement, i.e., comparable relative-differences (Stevens, 1946; Embretson, 2006).

Unfortunately, while the theoretical tenets of IRT have been published for nearly half- century, its incidence has been modest (Lord and Novick, 1968). An historical review of empirical methods in *Journal of Applied Psychology* indicated zero applications of IRT (Austin et al., 2002). The most recent review of studies published from 1997 to 2007 in *Organizational Research Methods* indicated a slight uptick to 3% (Aguinis et al., 2008). Grimm et al. (2013) echo the sentiment of these findings,

Often, the same measurement instrument is administered throughout a longitudinal study and the invariance of measurement properties is assumed. What often goes unrecognized in these situations is that the sum of item responses represents a specific measurement model—one where each item is weighted equally and interval-level measurement is assumed. (p. 504)

### Measurement Error

CTT assumes equal measurement error across all score levels (c.f., Feldt and Brennan, 1989). Concomitantly, because CTT typically only models total-scores, “items are considered to be parallel instruments” (van Alphen et al., 1994, p. 197). This additive (independent) treatment of measurement error likely holds serious implications for linear predictions made from disparate work and retirement research domains. Methodologically, it pits predictions to be diametrically opposed by sake of contrasting error distributions (Kerry and Embretson, 2018).

In contrast to CTT, IRT measurement error varies over the latent-trait distribution. It varies primarily as a function of score-information available, for example, (1) items with higher discriminatory power (loadings) generally have less error, and (2) items with locations (intercepts) nearer a population’s mean generally have less error.

Analogous to the principal of multilevel analyses performed by Weikamp and Göritz, multilevel tests is a measurement-based alternative. It would presume theoretical knowledge of population-differences for administering optimally scaled items. Without population- distribution knowledge, assessment would be feasible under adaptive administration (Lord, 1980) (c.f., Wainer, 1993). Unfortunately, the noted measurement errors from CTT’s scoring model is propagated by measurement design. This will be briefly addressed next in terms of change scores.

### Change Scores

Weikamp and Göritz report that approximately 1/3 of their sample (*N* = 718) constituted two-wave completions. This data quality is traditionally termed ‘difference’ scores (Guilford, 1954). Bereiter (1963) noted three particular challenges to interpreting difference scores, including, (1) spurious negative correlations with baseline standing, (2) differential meaning from baseline level, and (3) paradoxical reliability. The first issue is addressed in a later section with an IRT analysis. The latter two CTT-scoring issues will be more directly addressed below.

#### Differential Meaning From Baseline

In terms of differential change as a function of initial standing, this is largely due to confounding of fixed-item content and differential change of individuals over a fixed time-scale. That is, when item-difficulty (intercept) is poorly matched to the sample, little change will be detected based on observed-scores. An instantiation of this issue from Weikamp and Göritz is elaborated as an instructive example.

Weikamp and Göritz (2015, p. 374) report a significant ‘Age × Remaining Time’ cross-level interaction, such that, “younger adults exhibited a steeper decline in perceived remaining time across the 4 years than did older adults^3^. An alternative explanation may be that the observed-effect is due to scale-interval artifacts. That is, the differential appropriateness of test difficulty. The items in the ‘remaining time’ subscale are relatively difficult as indicated by the lower mean to median values (2.92 < 3) and, hence, more appropriate for detecting changes at higher levels of the latent factor. Because the mean-score for younger workers is substantially higher than that for older workers, *t_(312)_* = -35.75, *p* < 0.01, an apparently larger decrement is observed for analyses based on CTT scores.

The scaling artifact would compound with the design artifact of spurious-negative correlations associated with two-wave data, which is more prevalent among younger than older workers, *t_(312)_* = -6.64, *p* < 0.00. To put simply, the variety of difficulty parameters desirable for lifespan theory measures (representing spectrum of latent-trait levels) are equated in CTT-scoring and, consequently, contrives (biases) the study of age-differences, longitudinally or otherwise (see, Kerry and Embretson (2018) for experimentally pitted predictions originating from different lifespan theory origins of early childhood vs. gerontological).

#### Paradoxical Reliability

Bereiter (1963) noted that as correlations between measures increase, the reliability of their difference scores decrease.^4^ This principal (limitation) partially reflects in internal reliability estimates, as well. For example, based on greater average test–retest correlations for ‘remaining time’ (*r* = 0.74), compared to ‘remaining opportunities’ (*r* = 0.65), Weikamp and Göritz report that ‘remaining time’ is relatively more stable over time. In addition, however, the corresponding internal reliability estimates (*α* = 0.64-0.74) is lower than that for ‘remaining opportunities’ (*α* = 0.92–0.95). A statistical test based on the unweighted averages (0.70, 0.94 respectively) over all assessments indicated a significant difference, *X^2^_(1)_* = 917.31, *p* < 0.00. The more informative result depends on the reliability valuation of a given researcher.

More contradicting is the issue of individual item-scoring, in particular, the lower internal reliability estimate of ‘remaining time’ is likely partly owed to the inclusion of a reverse-scored item. To examine this issue possibility, matched two-wave data on the FTP instrument was obtained from RAND’s American Life Panel (ALP). Collection occurred from 2012 to 2014, with an average time-scale of (18 months) (*N* = 620).^5^ From this dataset, it was determined that the average temporal consistency for standard-scored items (*r* = 0.49) was significantly greater than that for reverse-scored items (*r* = 0.37), *z*(1) = 2.53, *p* < 0.01.

### Summary

This first section addressed some limitations to the analyses of change-scores based on CTT. The remainder of this commentary will utilize IRT measurement models for all analyses. Two substantive questions are addressed which have implications for the longitudinal assessment of O-FTP. The next section addresses the factorization of the original and adapted FTP instrument.^6^

## Chop

Here, it is argued that the methodological bifurcation of an instrument based on is tantamount to the substantive disintegration of work – retirement scholarship. First, I review prior evidence, and present new evidence, on the empirical justification for multiple-factor solutions to FTP. Beginning with Cate and John’s (2007)^7^ original exploratory study, it has long been known that measurement model-over specification (addition of latent factors) will typically lead to better model-data fit, though at the expense of sample fluctuations, regardless of correct measurement-model specification (MacCallum et al., 1992).

Improved model-data fit may be insufficient empirical justification for specifying additional latent factors, particularly amid current verisimilitude for work – retirement domain integration. Methodologically speaking, recruiting strong evidence for the latent-factor structure of FTP (by extension, O-FTP) is critical to its longitudinal study, because “changes in the number of latent variables would constitute violations of measurement invariance,” and “factorial invariance is a weaker condition than measurement variance” (Millsap et al., 2012, pp. 109–110). Specifically, factorial invariance is ‘weaker’ because it requires only conditional invariance of the mean and variance (Millsap, 2011). In other words, factorial invariance stops at conditional symmetry of the distributions. How does this information-limit substantively relate to work and retirement disintegration?

A more concrete and illustrative example may be found in the dichotomization of organizational and retirement scholars’ application of FTP for functionally dissimilar purposes. Specifically, organizational scholars (consistent with lifespan theorists) postulate *decreasing* age-related changes (Zacher and Frese, 2009), contrary, retirement scholars postulate *increasing* age-related changes (Hershey et al., 2010). In this case, two self-report instruments of FTP across functionally dissimilar populations (workers and retirees) vesseled weak validity evidence and poor verisimilitude (Cronbach, 1988; Meehl, 1990). Extrapolating, the substantive disconnect rationally led to contradictory predictions, i.e., asymmetry^8^, but as Cronbach and Meehl (1955) observed, “Rationalization is not construct validation” (p. 291).

### A Closer Look at the Measurement Model

In the previous section, I noted that improved model-data fit may be insufficient criterion for justifying the ‘factorization’ of FTP from its theoretical unidimensionality (Carstensen and Lang, 1996, Unpublished). Recently, a “rediscovery” of bifactor modeling has proved useful for accommodating the reality of multidimensional data (interested readers are directed to Reise, 2012). It provides a stronger empirical criterion for justifying latent-factor structure of instruments. For example, McKay et al. (2015) successfully applied the bifactor model toward resolving contradictory reports on the latent-structure of the *Consideration of Future Consequences* instrument. The authors concluded, “conceptual utility cannot be at the expense of measurement accuracy” (p. 6).

Regarding FTP, a bifactor model-comparisons approach was recently adopted using data-fit indices. Similar to McKay et al.’s (2015) findings, application of the bifactor model (*N* = 2,185) resulted in support for retention of the bifactor solution, relative to the previously reported two-factor structure (Kerry, 2017). Also, additional analyses failed to find support for meaningful interpretation of subscale scores (Haberman, 2008).

Turning to FTP’s adaptation, in the initial dissertation study on which the O-FTP instrument is based, 6/10 original FTP items were retained following an exploratory factor analysis (EFA). Though unstated, exclusion was presumably because of high cross-loadings (*Λ*j > 0.30), resulting in three items each representing the two subscales that have been used in subsequent O-FTP studies. Despite the exclusion of high cross-loading items (Little et al., 1999; Smith et al., 2000), a non-negligible correlate of *r* = 0.69 was reported between the two subscales (Zacher and Frese, 2009). In order to better determine the “essential dimensionality” of the O-FTP instrument (Stout, 1990), the next section extends evidence from bifactor modeling of FTP to O-FTP data.

### Bifactor Modeling of O-FTP

In a mixed-age (22 – 60-years) sample of working adults, a model-comparison was conducted on the O-FTP instrument (*N* = 511). First, a unidimensional model was estimated as a baseline restricted-model. Second, a multidimensional (2-correlated factors) model was estimated in replication of Weikamp and Göritz’s measurement model. Third, a bifactor model was estimated whereby all items loaded on a common factor and two orthogonal facets. The results are reported in **Table 1** below. As expected, the two-factor solution exhibited greater model-data fit relative to the unidimensional model, though only according to information-criteria (*-2lnL, AIC, BIC*), while the residual-based criterion (RMSEA) indicated comparatively worse fit. In addition, the bifactor solution exhibited greater model-data fit relative to the two-factor solution, *X^2^_(5)_* = 24.75, *p* < 0.00, without a concomitant increase in model error as indicated by RMSEA. Using a model-comparison approach, these findings extend support for the bifactor solution to FTP data to the adapted, O-FTP instrument.

**Table 1:** Comparative model-data fit indices for O-FTP.

Model	-2/*nL* (*df*)	AIC	BIC	RMSEA
1-Dim	8672.77 (*534*)	8756.77	8934.70	0.10
2-Dim	8588.96 (*533*)	8674.96	8857.12	0.12
Bifact	8554.21 (*528*)	8650.21	8853.56	0.12

*N = 511. -2lnL, -2 log likelihood; AIC, Akiake information criterion; BIC, Bayesian information criterion; 1-DIM, unidimensional model; 2-Dim, two-dimensional model; Bifact, unidimensional bifactor model. For 2-Dim model, estimated 𝜃12 = 0.87.*

In order to complement the model-comparisons approach and better examine the potential dimensionality-distortion in the O-FTP instrument, a direct-modeling procedure was used to compare item-factor loading patterns across unidimensional and bifactor models. Results in **Table 2** indicate negligible differences in the factor- loading patterns. These findings suggest minimal distortion of structural parameter estimates from fitting a unidimensional measurement model to the multidimensional data.

**Table 2:** Summary item-factor loading patterns across unidimensional and bifactor estimated models.

Item	Uni-Dim λ	Bifactor λ_General_
O-FTP 1	0.90	0.89
O-FTP 2	0.91	0.89
O-FTP 3	0.96	0.96
O-FTP 4	0.84	0.83
O-FTP 5	0.70	0.65
O-FTP 6	0.67	0.66

*N = 511.*

Taken together, the findings suggest that the bifactor model should become integral to the model-comparisons approach when justifying latent dimensionality of an instrument based solely on model-data fit indices. The findings for better model-data fit with specification replicate those obtained by Weikamp and Göritz. Indeed, the authors confer an understanding of the “trade-off between fit and parsimony” (p. 375) for selecting their base model for multilevel-analytic comparisons. The current analysis merely complements the application of this principle when specifying a baseline-measurement model, presumably as precedent to longitudinal analysis.

## Change

At the outset of this commentary, I noted the statistical-equivalence of procedures for assessing measurement invariance over time and across groups (Meredith, 1993). In the previous section, I addressed the factor-structure of FTP and O-FTP in cross-sectional data with the bifactor model. In this section, I continue with the ‘statistical-equivalence’ framework with an instructive application of a longitudinal extension of the bifactor model. Importantly, this model builds on the observation of Embretson (1991, p. 511) to, “conceptualize change as a separate dimension” by extending such conceptualization to the item- level. This is also important from a measurement design perspective, because CTT scores are typically derived from fixed-content forms, incurring practice effects to the propagation of measurement error. Put simply, practice effects will confound time effects.

In order to better account for the utility of modeling item-response dependence over time, a unidimensional longitudinal-model will be fit as a comparator. One notable departure from prior terminology of measurement invariance, in IRT application, measurement invariance is typically termed differential item functioning (DIF). DIF may be defined as differences in parameters of item-response functions across groups or over time (Thissen and Wainer, 2001). Analyses were conducted on the same two-wave FTP data that was used in the first section (*N* = 620).

### Uni-dimensional Longitudinal Model

Likelihood- ratio based statistics for the unidimensional-fitted model are reported in **Table 3** below. Specifically, **Table 3** displays values from the overall-DIF statistics decomposed into discrimination (slope) and location (intercept) parameter estimates. Three items exhibited evidence of systematic DIF at nominal levels of statistical significance. Latent-mean estimates indicated almost no change in the level of FTP, while variability slightly increased (*𝜃-*μT2 = 0.01, *𝜃-*σT2 = 1.07). It should be noted that these findings generally accord with the first section’s treatment of temporal reliability of reverse-scored items.

**Table 3:** Summary Uni-DIF statistics by slope and location parameter estimates for time.

Item	*X*^2^_(location)_	*Df*	*p*-value	*X*^2^_(slope)_	*Df*	*p*-value
FTP 1	9	6	0.17	0.2	1	0.68
FTP 2R	17.8	6	0.01	0.5	1	0.47
FTP 3	3.9	6	0.68	0	1	0.92
FTP 4	1.8	6	0.94	0.2	1	0.67
FTP 5	4.8	6	0.57	0.2	1	0.69
FTP 6	6.7	6	0.35	0.5	1	0.48
FTP 7	7	6	0.32	3.2	1	0.07
FTP 8	5.3	6	0.50	2.6	1	0.11
FTP 9R	14.3	6	0.03	0.4	1	0.52
FTP 10R	13.8	6	0.03	0.6	1	0.44

*N = 1,240. Anchored on all items.*

### Longitudinal Bifactor Model

**FIGURE 1:**
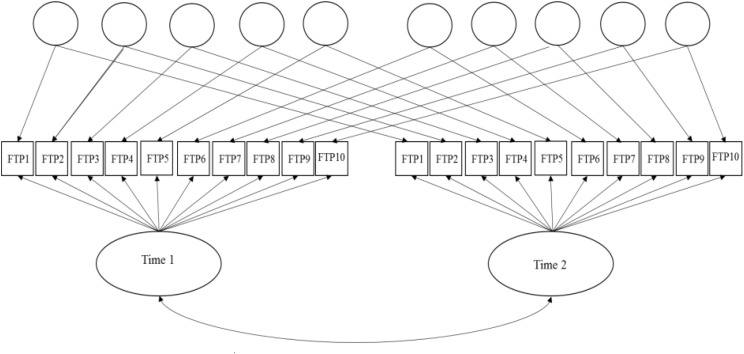
Graphical representation of two-tier model for FTP longitudinal item response data.

In order to better account for lack of conditional independence owed to specific item parameter estimates and time in this single-group, common-items design, a longitudinal adaptation of Cai’s two-tier full-information bifactor model is estimated (see, **Figure 1**, also, Yin, 2013, Unpublished).^9^ The longitudinal bifactor model comprised two primary factors and ten specific factors (one per item) (Hill, 2006, Unpublished). Primary factors represent the measured latent construct at each assessment (time 1 and 2). The specific factors (item doublets over time) capture the lack of conditional independence, that is, item-level correlated residuals over time. After imposing identification equality-constraints (see Cai, 2010 for details), the mean of the second primary dimension (time 2) is estimated and represents latent-change (level) in FTP from time-1 to time-2. Additionally, the covariance between primary dimensions may be estimated and represents the stability of the latent construct over time. Parameter estimates and model fit indices are reported in **Table 4** below.^10^

**Table 4:** Longitudinal two-tier full-info FTP item bifactor analysis over approximately 2 years.

		Intercepts						Primary factors		Specific factors										
				
Time	Item	1	2	3	4	5	6	Time 1	Time 2	1	2	3	4	5	6	7	8	9	10	
1	1	6.08	4.03	2.3	0.26	-1.47	-3.14	3.22 (0.19)		2.12										
1	2	3.35	2.34	1.38	0.36	-0.51	-1.83	1.42 (0.09)			0.89									
1	3	5.99	3.44	1.81	-0.39	-2.12	-3.8	2.91 (0.18)				1.09								
1	4	8.29	5.18	3.22	0.74	-1.59	-3.77	4.36 (0.38)					0.72							
1	5	3.52	1.05	-0.74	-2.8	-4.28	-5.57	2.61 (0.17)						1.87						
1	6	2.94	1.03	-0.22	-1.85	-3.16	-4.33	2.11 (0.14)							1.39					
1	7	4.52	2.4	1.07	-0.5	-2.09	-3.55	2.03 (0.13)								1.31				
1	8	4.22	2.13	0.78	-0.81	-2.26	-3.96	1.92 (0.12)									0.96			
1	9	3.56	1.99	0.86	-0.28	-1.14	-2.57	1.08 (0.09)										1.29		
1	10	2.44	0.84	-0.36	-1.56	-2.48	-3.94	0.89 (0.09)											1.49	
2	1	6.08	4.03	2.3	0.26	-1.47	-3.14		3.22 (0.19)	2.12										
2	2	3.35	2.34	1.38	0.36	-0.51	-1.83		1.42 (0.09)		0.89									
2	3	5.99	3.44	1.81	-0.39	-2.12	-3.8		2.91 (0.18)			1.09								
2	4	8.29	5.18	3.22	0.74	-1.59	-3.77		4.36 (0.38)				0.72							
2	5	3.52	1.05	-0.74	-2.8	-4.28	-5.57		2.61 (0.17)					1.87						
2	6	2.94	1.03	-0.22	-1.85	-3.16	-4.33		2.11 (0.14)						1.39					
2	7	4.52	2.4	1.07	-0.5	-2.09	-3.55		2.03 (0.13)							1.31				
2	8	4.22	2.13	0.78	-0.81	-2.26	-3.96		1.92 (0.12)								0.96			
2	9	3.56	1.99	0.86	-0.28	-1.14	-2.57		1.08 (0.09)									1.29		
2	10	2.44	0.84	-0.36	-1.56	-2.48	-3.94		0.89 (0.09)										1.49	

						Factor means		0.00	-0.02 (0.04)											
	
						Primary factor variances		1.00	1.11 (0.09)	Specific factor variances										
	
						Covariance matrix		0.70 (0.04)		1.00	1.00	1.00	1.00	1.00	1.00	1.00	1.00	1.00	1.00	1.00

						Model fit		M^2^ (*df*)		-2*ln*L			AIC			BIC			RMSEA	

								644.49 (*127*)		40273.23			40439.23			40806.9			0.08	

*Fixed (zero) slopes shown as blank entries. Standard errors in parentheses. N = 620-matched participants.*

Similar to the unidimensional model, latent mean-level change in FTP was negligible (*𝜃-*μT2 = -0.02) and variability increased only slightly (*𝜃-*σT2 = 1.11). The latent-stability estimate from the covariance matrix is fairly high at *σ*2,1 = 0.70. All primary factor slopes (loadings) are strong and significant, as well as the specific factor slopes (loadings).

Given earlier arguments against overfitting of measurement models, a precautionary comparison for this more complex model seemed warranted. Specifically, in order to determine whether item-level residual dependence need-be accounted for when estimating latent stability, a two-dimensional model without item doublets was estimated (2-Dim). The likelihood-ratio comparison between these two nested models is highly significant (*X^2^_10_* = 759.46, *p* < 0.001), suggesting that item-level residual dependence should not be ignored.

## Discussion

The current commentary was methodologically motivated, but with substantive purpose (Pedhazur and Schmelkin, 1991). Weikamp and Göritz conducted a valuable longitudinal study, and they deployed admirably sophisticated statistical analyses. This commentary aimed to complement these efforts with attention to measurement in the research context of aging populations. Three sections addressed a variety of measurement issues, summarized below.

In the first section, I overviewed some of the limitations of analyses and inferences drawn from statistical models applied to CTT-based measures. Choice of test theory model (CTT vs. IRT)^11^ and respective implications for measurement error was noted. Two concomitant examples of CTT-based measurement error were emphasized in the context of change scores: (1) comparability of differential baseline scores, and (2) paradoxical reliability.

In the second section, *Chop*, I overviewed the empirical justifications for rescoring the original FTP as a two-factor structure, noting the insufficiency of model-data fit indices and vulnerability to sampling variability. An instructive example with opposing age-related predictions for FTP across work and retirement domains was presented. The bifactor measurement model was introduced as a more integral, empirical justification of latent-factor specification. Recent evidence of an optimal bifactor solution for FTP data was extended to the O-FTP instrument, supporting the retention of a unidimensional structure.

In the third section, *Change*, the measurement design (fixed content) of CTT-based scores was noted for introducing potential practice effects as an additional source of measurement error when assessing change. A longitudinal extension of the bifactor model (two-tier) assessed the influence of item-level residual dependencies over time, indicating that they should be accounted for in fixed-content, repeated-measures designs.^12^

### Substantive and Theoretical Considerations

Having devoted considerable space to methodology, there are a couple noteworthy substantive and theoretical considerations. First, content-wise, some of the item design features of the FTP instrument may be reifications of the work – retirement disjunction itself. For example, FTP item features primarily conflate two historical conceptualizations of ‘cognitive extension’ (Wallace, 1956) and ‘future affectivity’ (Hooper, 1963, Unpublished). More generally, the relative impact of work – non-work valuation (affect) and short – long time horizons (cognitive) as common causes to work and retirement has not yet been comprehensively addressed. This accords with Wang and Shultz’s (2010) observation from their review of psychological paradigms of retirement research, “…very few studies that examined outcomes of retirement have incorporated factors that influenced the original retirement decision…This creates a logic gap because the reasons why people decide to retire would naturally influence how they evaluate outcomes associated with their retirement” (p. 176).

It may also be helpful to begin calibrating temporal research designs with focal constructs and attendant theories. For example, Ram and Grimm (2015) recently outlined a taxonomy of change processes from lifespan theory conceptions, with three heuristic examples of: (1) incremental, (2) transformational, and (3) stability-maintenance. Socio- emotional selectivity theory, of which FTP is a “cardinal tenet” (Carstensen et al., 1999, p. 167) may be most accurately associated with ‘incremental’ change processes. However, the original adaptation of FTP to workspace (Occupational-FTP) consistently characterizes the construct as “state-like” (Zacher and Frese, 2009, p. 148). The distinction is important, because state-like conceptualizations favor stability-maintenance change process models, which generally concerns *intra*-individual variability, registered on smaller time-scales, and with more frequent assessments (e.g., experience sampling, sensory data, etc.). In contrast, Weikamp and Göritz’s 4-year study is a fairly moderate-large timescale for human lifespan. In short, in as much as worklife is subordinate to biologic life, a change in focal construct conceptualization has implications for the optimal change-process model that is applied (c.f., Ekerdt, 2004).

More substantive, how does O-FTP accord with shifts in labor relations, e.g., job mobility and psychological contracts? Would O-FTP show expected variations as a function of, say, occupational hazards? Can earlier SST findings for FTP generate plausible rival hypotheses with O-FTP vis-à-vis other job features (e.g., employer-sponsored health insurance)? It is a *non sequitur* that occupational-FTP is necessarily indicative of career aspirations amid increasing life expectancies. Consider how the concurrency of work- recovery cycles may complement the continuity of phased-workforce withdrawal. In short, the concurrent changes in work and retirement cannot be reduced to a mere cohort effect, rather, they are functionally interdependent with the goal of optimizing any individual’s given time.

### Closing Thoughts

In principal, industrial-organizational psychologists provide expertise for evaluating the quality of individual difference measures. In practice, we are behooved to utilize design, measurement, and analysis as quantitative informants for our research topics. To the extent that age-integration of social institutions and domain-integration of work-retirement continues, we will likely be better guided by more equitable approaches.

## Author Contributions

The submitting author scoped the focal article for instructive exemplification, conducted analyses, and contributed all expository and technical aspects of the paper’s write-up.

## Conflict of Interest Statement

The authors declare that the research was conducted in the absence of any commercial or financial relationships that could be construed as a potential conflict of interest.

## Footnotes

1 Note, “data analysis" as distinct from “statistical analysis" (Tukey, 1986).

2 Unfortunately, statistical standardization is ineffective for solving the measurement-scaling artifact because it is bound by linearity.

3 Apropos to the current commentary, the highly statistically significant (*p* < 0.001) effect estimate for this interaction is a whopping *β* = 0.0002 with *SE* = 0, which is owed to the report delimitation of three decimal places, except for the effect estimate where it is extended to four.

4 Conversely, an increase in ‘difference-score’ reliability from low test–retest correlations introduces difficulty in interpreting the meaning of ‘change,’ as it would imply the test does not measure the same latent construct. Embretson (1991) explained the seeming paradox stems from failure to conceptualize and model ‘change’ as a separate dimension, which is resolved through the application of an item-response model.

5 More details regarding the original sampling source used for the current article’s tutorial-demonstration purposes can be found online at →https://www.researchgate.net/profile/Matthew_Kerry/contributions.

6 In **Appendix A**, a presentation for identifying temporal outliers according to test–retest designs is presented for interested readers.

7 Foregoing additional methodological concerns, such as the truncation of response-options (from 7- to 3-point Likert-type scales), combined with the use of Pearson (rather than polychoric) correlations, which typically results in reduced item inter-correlations.

8 Along with poorly specified nomological networks within work and retirement domains, which perhaps would have qualified as “strong programs” of research permitting perhaps plausible, rather than incidental, rival hypotheses (Campbell, 1960; Cronbach, 1989).

9 It should be noted that this measurement model is conceptually similar to Fischer’s (1995) Rasch-based linear logistic model for change.

10 It should be noted that the analytic-model is flexible to longitudinal (>2-wave) designs that would relax some identifying-equality constraints.

11 It should be noted that these are not exhaustive test theory models, e.g., generalizability theory (Cronbach et al., 1963).

12 Interested readers who were earlier-directed to **Appendix A1** for introduction to the D_ptc_ index for detecting ‘temporal outliers’ are further encouraged to read **Appendix A2** here. In **Appendix A2**, specifically, the longitudinal bifactor model was re-estimated after removal of ‘temporal outliers’ based on the statistic introduced in preceding **Appendix A1**, resulting in substantially greater stability estimates.

## Appendix A

### Demonstrative Application of Novel CTT-Based Tool (D_ptc_)

#### (A1) Practical tool *(Temporally inconsistent responders)*

There may be many reasons to expect score-fluctuations in repeated-measures designs (Thorndike, 1947). Incumbent on all social scientists working with longitudinal design data is the identification of erroneous response patterns, for example, insufficient-effort responding (IER; Huang et al., 2012). In addition to item-design features (validation items, true-effort solicitation) and para-data (response times), numerous *post hoc* statistical procedures have also been developed.

Recently, an inventive adaptation of two conventional statistics for identifying IER was developed on simulation data (see, DeSimone, 2014). It conceptually integrates principles from Jackson’s (1976) personal reliability coefficient and the more-familiar, Mahalanobis distance score (*D*; Mahalanobis, 1936). A full account of its computation is beyond the scope of this study, but its’ central premise rests on replacing ‘centered’ values in the original Mahalanobis-D formula with ‘individual difference scores’ between two time points. For further information, readers are referred to DeSimone (2014), but its formula is given below,

$D_{ptc}=\sqrt{{\left(\vec{\underset{i\left(t1\right)}{X}}-\vec{X_{i\left(t2\right)}}\right)}^{T}DIF_{xx}^{-1}\left(\vec{\underset{i\left(t1\right)}{X}}-\vec{X_{i\left(t2\right)}}\right)}$

where *Xi(t1)* and *Xi(t2)* indicate theresponse vectors for participant *i* at times (t1) and (t2), respectively. *DIF^-1^* is defined as the inverted covariance matrix with difference-scores. As a multivariate distance indicator based on raw response patterns over time (*and* within- individual), *Dptc* could become a powerful tool for strengthening the quality of longitudinal data. As with the original Mahalanobis-D, *D^2^* is asymptotically distributed as chi-square statistic, permitting statistical tests of significance with degrees of equal to test items.

Applying this formula to the current two-wave FTP dataset, *N* = 94 respondents were flagged with a significant D_ptc_ value, *X^2^_(10)_* = 18.31, *p* < 0.05. After removal, as expected, the average temporal consistencies increased for, both standard-scored (*r* = 0.49 → 0.59) and reverse-scored items (*r* = 0.37 →0.44).^13^ Though not previously addressed, it was considered what impact, if any, this exclusion criterion might have on corresponding internal reliability estimates. In order to link the analyses back to Weikamp and Göritz, as well as inform practical utility, average-Cronbach alphas and item-level test–retest correlations were computed for the three items, each, corresponding to the ‘remaining opportunities’ and ‘remaining time’ subscales. Results of this analysis are reported in **Table A1** below.

**Table A1:** Summary internal and temporal reliability estimates by samples and subscales.

	Remaining Opps		Remaining Time	
		
	$\left(\vec{X}\right)$	$\alpha \left(\vec{X}\right)Test-retest$	$\left(\vec{X}\right)$	$\alpha \left(\vec{X}\right)Test-retest$
Full (*N* = 620)	0.89	0.48	0.71	0.49
Stable (*N* = 526)	0.90	0.56	0.74	0.59

*X^2^_(10)_ = 18.31 value used (p < 0.05) for identifying temporally inconsistent responders.*

Interestingly, the scale-based internal reliability estimates did not suffer from the same decrement as a result of removing ‘temporally inconsistent’ responders. Given that this is the first empirical application of the newly developed statistic, the current author endorses the use of the multivariate tool for identifying IER in longitudinal datasets, particularly as a viable observed-score solution.

#### (A2) Exploratory D_ptc_ application extensions

In interest to extend practical evidence for the ‘temporal inconsistency’ statistic (D_ptc_) from the first section, the three models in **Table A2** were re-estimated on a reduced sample, according to removal of significant D_ptc_ values (*N* = 94). For clarity, only fit-indices for the bifactor model are again-reported in **Table A2**, but it should be noted that all fit indices (including information criteria) indicated analogously better model-data fit with the reduced sample. Substantive departures from initial findings are summarized below, in order, for each model.

**Table A2:** Comparative fit indices for longitudinal models.

Model	1M^2^ (*df*), *p*-value	-2*ln*L	AIC	BIC	RMSEA
1-Dim	9637.47 (3220), *p* < 0.00	40917.53	41199.53	41921.85	0.04
BiFact	644.49 (127), *p* < 0.00	40273.23	40439.23	40806.90	0.08
2-Dim	1304.21 (137), *p* < .00	41032.69	41178.69	41502.06	0.12
Reduced	600.19 (127), *p* < 0.00	32801.60	32967.60	33321.62	0.08

*N = 1,240. -2lnL, -2 log likelihood; AIC, Akiake information criterion; BIC, Bayesian information criterion; 1-Dim, Unidimensional model; Bifact, longitudinal bifactor model; 2-Dim, two-dimensional model; Reduced, Dptc-reduced sample with longitudinal bifactor analysis.*

Discrepant from the first model, one of the three items that formerly exhibited systematic DIF (reverse-scored) became non-significant. From the longitudinal bifactor model, mean-FTP and variability was similar to the original sample (both, negligible change), however, the latent-stability estimate from the covariance matrix increased, from *σ*2,1 = 0.70 to 0.80. Also noteworthy, the *RMSEA* was maintained at 0.08, despite removal of approximately 15% of the sample. Finally, from the two-dimensional model, the latent-stability estimate further increased to *σ*2,1 = 0.84, which coincided with increased model-error, *RMSEA* = 0.11. This is, perhaps, a straightforward demonstration of how stability-estimates may be inflated, but relative to the measurement model error.

Now, recall from the first section, whereby removal of the D_ptc_-flagged (temporally inconsistent) respondents resulted in higher item-level test–retest correlations, but did *not* reduce Cronbach’s α (scale-level)? This finding is extended, here, in principle. That is, neglecting to model item-level error terms over time (2-Dim) led to increased stability estimates, but at the expense of greater measurement model error and comparatively worse fit. This is owed to the fact that, model-data fit increases as, (1) predictors are added (e.g., 2-factor vs. 1-factor), and (2) sample size decreases.

Unfortunately, the discrepant measurement error is not accounted for, or ignored, in statistical analyses applied to CTT-based measurement models. In simplest terms, CTT-measurement assumptions impose information-loss via equating of item-error and, subsequently, uniform test-level error across all levels on the latent continuum. In their review of measurement issues when studying change, Edwards and Wirth (2009) remark,

“There is indeed something odd about the common practice of using factor analysis to establish the dimensionality of a scale but then ignoring the parameter estimates themselves when creating scale scores. Statements about the adequacy of a model from a factor analytic standpoint may not apply when the parameters from that model are ignored” (p. 84–85).

I agree, but I might also clarify as to ‘adequacy’ and ‘applicability.’ In refutation, two-factor structures (more predictors) are justified by greater model-data fit. Subsequently, FTP is dichotomized (inter-factor correlations set to zero) when used in prediction equations. This is information-loss. From a sampling perspective, this may be akin to the information-loss associated with median-splits. From a substantive perspective, this may reflect in artefactual divides between work and retirement research. I advocate the combined use of the newly developed D_ptc_ statistic and model-based measurement to recover optimal information for a problem space. As Kerlinger and Pedhazur (1973) cautioned of ‘mere’ statistics, “There is a curious mythology about understanding…the technical aspects of research…many behavioral researchers say they will use a statistician and a computer expert to analyze their data. An artificial dichotomy between problem conception and data analysis is set up” (p. 368).
